# Impact of heating on passive and active biomechanics of suspended cells

**DOI:** 10.1098/rsfs.2013.0069

**Published:** 2014-04-06

**Authors:** C. J. Chan, G. Whyte, L. Boyde, G. Salbreux, J. Guck

**Affiliations:** 1 Cavendish Laboratory, Department of Physics, University of Cambridge, Cambridge, UK; 2 Biotechnology Center, Technische Universität Dresden, Dresden, Germany; 3 Department of Physics and Institute of Medical Biotechnology, University of Erlangen-Nuremberg, Erlangen, Germany; 4 Max Planck Institute for the Physics of Complex Systems, Dresden, Germany

**Keywords:** cell contraction, calcium, cortical tension, myeloid precursor cell, optical stretcher, cell rheology

## Abstract

A cell is a complex material whose mechanical properties are essential for its normal functions. Heating can have a dramatic effect on these mechanical properties, similar to its impact on the dynamics of artificial polymer networks. We investigated such mechanical changes by the use of a microfluidic optical stretcher, which allowed us to probe cell mechanics when the cells were subjected to different heating conditions at different time scales. We find that HL60/S4 myeloid precursor cells become mechanically more compliant and fluid-like when subjected to either a sudden laser-induced temperature increase or prolonged exposure to higher ambient temperature. Above a critical temperature of 52 ± 1°C, we observed active cell contraction, which was strongly correlated with calcium influx through temperature-sensitive transient receptor potential vanilloid 2 (TRPV2) ion channels, followed by a subsequent expansion in cell volume. The change from passive to active cellular response can be effectively described by a mechanical model incorporating both active stress and viscoelastic components. Our work highlights the role of TRPV2 in regulating the thermomechanical response of cells. It also offers insights into how cortical tension and osmotic pressure govern cell mechanics and regulate cell-shape changes in response to heat and mechanical stress.

## 1. Introduction

Cells exhibit a rich dynamic behaviour when subjected to mechanical stress. Over the years, several theoretical approaches have been developed to describe cell mechanical properties, although there is no unambiguously accepted theoretical description as of yet. These approaches range from phenomenological models that picture cells as a suitable combination of spring and dashpot elements [1,2] to the scale-free power-law models that well characterize the physical stress response of a large class of living cells [3,4]. To this end, a vast array of experimental techniques have been developed to study cellular mechanics. For example, magnetic twisting cytometry and atomic force microscopy [5–7] have been used extensively to probe local stress response of adherent cells, while micropipette aspiration and microplate manipulation have been developed to assess global mechanical properties of cells [8–10]. Comprehensive reviews of the different techniques can be found in recent publications [11–13]. Among these techniques, the microfluidic optical stretcher has emerged as a standard tool to probe whole-cell mechanics in a non-invasive way [14], hence offering powerful insights into the dynamics of non-adherent cells, for example during amoeboid migration [15,16].

Despite a great deal of research on cell mechanics, little is known about the impact of heating on cells. Previous studies [17–19] showed that both adherent and suspended cells, such as fibroblasts and leucocytes, soften with increased temperature. By contrast, other studies [20] suggested that adherent cells become stiffer and more solid-like with increased temperature, possibly owing to increased contractile activity of molecular motors. The exact mechanisms for such temperature-induced cell stiffening or softening remain elusive. In a similar vein, the impact of heating in cell deformation studies with an optical stretcher has been given little attention. Recent studies using fluorescence ratio thermometry [21] showed that at a wavelength of 1064 nm, which is most often used for optical trapping, 1 W of laser power will induce a heating of about 13°C within a fraction of a second. Higher wavelengths will be absorbed even stronger and lead to more heating, roughly linearly proportional to the change in absorption coefficient of water. Further work quantified the impact of laser heating on cell viability and calcium influx in an optical stretcher [22,23]. A recent study [24] provided further insights into how different time scales of heating may elicit different cell mechanical responses in an optical stretcher.

This paper therefore attempts to shed new light on the topic of temperature-induced changes of cell mechanical properties by measuring suspended cells in an optical stretcher when subjected to heating at various temperatures and time scales. Integrating the microfluidic optical stretcher with a 1480 nm heating laser and a heating chamber equipped with an adjustable heat source, we observed and characterized the degree of thermal softening of cells in the passive viscoelastic regime. Interestingly, we report striking evidence of active cell contraction whenever the overall temperature, independent of the combination of chamber temperature or laser-induced temperature increase, rose above 52°C. Such behaviour is correlated with a calcium influx regulated by temperature-sensitive transient receptor potential vanilloid 2 (TRPV2) ion channels. We showed that such a rheological transition from thermal softening to active cell contraction could be effectively modelled with a mechanically equivalent circuit. Our findings highlight the interesting role of TRPV2 ion channels in regulating cell mechanical properties and cell-shape changes in response to external heat and mechanical stress.

## 2. Experimental methods

### 2.1. Optical stretcher set-up

**Figure 1.**
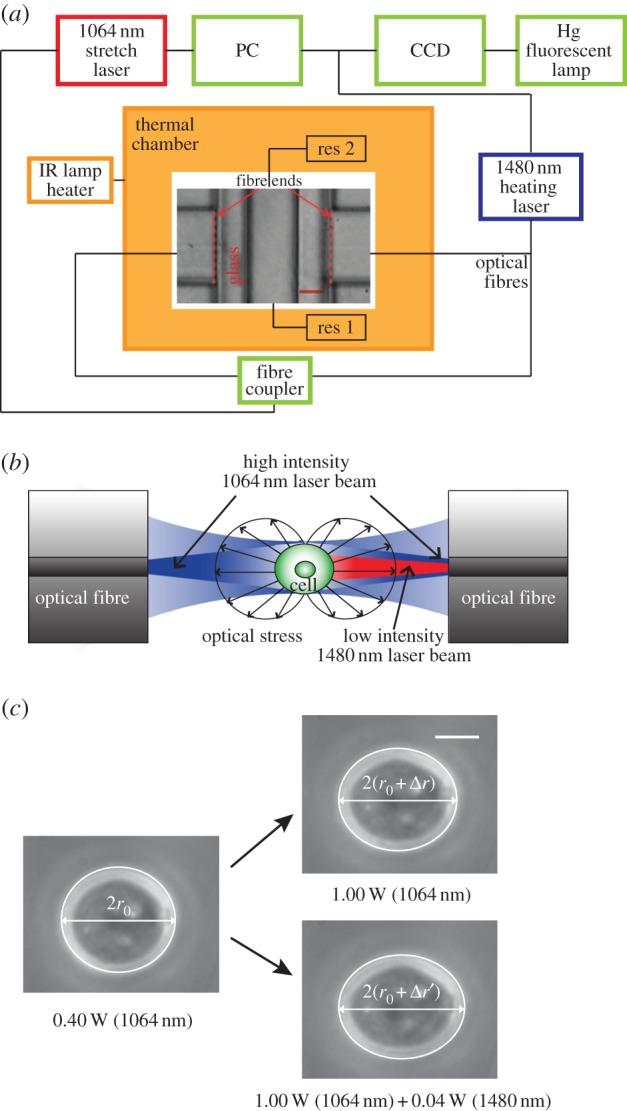
Experimental set-up of a microfluidic optical stretcher. (*a*) Schematic diagram of a microfluidic flow chamber (phase-contrast image) housed in a heating chamber, with optical fibres (left and right) arranged perpendicular to the walls of a square glass capillary (top to bottom), which contains cell suspension. Scale bar is 40 μm. (*b*) The momentum transfer of light to the surface of the cell results in (mainly axial) optically induced surface stresses, leading to the deformation of the cell along the optical axis. In addition to the two counterpropagating trapping and stretching laser beams (1064 nm, blue), an additional 1480 nm laser beam (red) was coupled into one of the optical fibres and co-propagated to irradiate and heat the cell with minimal additional power. (*c*) Left: an HL60 cell with radius *r*_0_ was trapped with a total power of 0.40 W in the optical stretcher. Right (top): an increase in the 1064 nm laser powers to 1.00 W led to a stretching of the cell along the laser axis, resulting in an increased radius 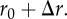 The strain *γ*(*t*) is then given by 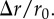 Right (bottom): applying 40 mW of 1480 nm heating laser led to a further axial strain 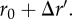 Scale bar is 5 μm.

The principle and set-up of the microfluidic optical stretcher have been described extensively elsewhere [25,26]. Essentially, the device is a dual beam laser trap capable of trapping and deforming the cells through optically induced stress acting on the cell surface. The microfluidic flow chamber, as shown with a phase-contrast image in figure 1*a*, consists of two co-axial optical fibres aligned perpendicular to a square glass capillary, which delivers the cells in suspension. The chamber is then mounted on an inverted phase-contrast microscope, which is attached to a CCD camera for image acquisition. The flow of cell suspension was adjusted through the relative difference in heights of inlet and outlet reservoirs connected to the capillary. The entire set-up was then enclosed in a heating chamber with the chamber temperature controlled by the use of an infrared lamp and heater. This allowed the cells to have sufficient time (about 30 min) to adapt to the new chamber temperature before being optically trapped and deformed.

In addition, the output of a laser diode operating at *λ*_0_ = 1480 nm was spliced into one of the 1064 nm optical fibres using a wavelength multiplexer, so that it can be operated simultaneously with the 1064 nm fibre laser. The coupling of 1480 nm single beam to one of the 1064 nm dual beams provides almost instantaneous (within milliseconds) heating in addition to cell deformation (figure 1*b*). As the temperature increase scales linearly with absorption coefficient at a particular wavelength, the about 180 times higher absorption at 1480 nm compared with 1064 nm [27] means that the 1480 nm laser could be used to introduce significant heating at minimal additional laser power, and consequently a negligible amount of optically induced stress on the cells. In this way, heating and stretching can be effectively decoupled. In contrast to adjustments of the chamber temperatures, this approach allowed us to probe temperature-dependent cell rheology when the cells were heated over a much shorter time scale. As shown in figure 1*c*, a cell can be effectively trapped and stretched with the 1064 nm stretch laser, with the axial strain readily obtained for further analysis as discussed in §2.2. Further application of the 1480 nm heating laser led to a further change in cellular strain, which demonstrates the working principle of the 1480 nm heating laser.

The laser-induced temperature increase inside the glass capillary was measured using fluorescence ratio thermometry, as described previously [21]. The method is based on measuring the laser-induced fluorescence of a highly temperature-sensitive dye (Rhodamine B) and a reference dye (Rhodamine 110) with temperature-independent fluorescence. The ratio of the intensities of the two dyes scales linearly with temperature. By varying the chamber temperature between 25 and 55°C, the intensity ratio was measured and converted into temperature values using the established calibration. This allowed us to obtain a precise measurement of the peak temperatures in the optical trap when the 1064 nm and the 1480 nm lasers were operated (see electronic supplementary material, figure S1). The increase in temperature for 1064 nm stretch laser and 1480 nm heating laser was measured to be 10.5°C W^−1^ and 2.2°C dBmW^−1^, respectively, where the power *P*_dBmW_, measured in decibels of power and the power *P*_W_, measured in W, are related by the equation: *P*_dBmW_ = 10log_10_(*P*_W_)+30.

### 2.2. Imaging and data analysis

A custom written Labview software was used to track the temporal evolution of the cell edge (figure 1*c*). Data for major axis deformation *r*(*t*) were stored for every time-frame, while *r*_0_ was evaluated as the mean length of major axis during the initial trapping period. The time-varying axial strain 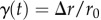, as depicted in figure 1*c*, was then evaluated accordingly. The optically induced stress was computed based on the generalized Lorenz–Mie model [28]. Once the peak stress *σ*_0_ along the laser axis was evaluated, the time-dependent creep compliance *J*(*t*) could be readily obtained2.1
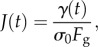
where *F*_g_ is a geometric factor that takes into account the cell size and stress distribution [29].

Recent studies have shown that one can employ either the power-law or the mechanical models to extract cell viscoelastic parameters in the optical stretcher experiments [4,16,30]. The equation for the power-law model is as follows:2.2
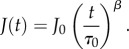
Here, a value of *β* = 0 gives Hooke's law while *β* = 1 corresponds to complete viscous behaviour. *τ*_0_ = 1 s and *J*_0_ is defined as the characteristic inverse Young's modulus. *β* is, therefore, a measure of the cell fluidity while *J*_0_ is a measure of cell compliance. On the other hand, a simple mechanical model, for example the standard linear liquid (SLL) model, has been recently shown to characterize cell deformation for blood precursor cells [16]. The model employs the following equation for the creep compliance:2.3
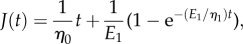
where *η*_0_ describes the steady-state viscosity of the model, *η*_1_ and *E*_1_ the transient viscous and elastic response, respectively.

In estimating changes in cell volume, we note that the tracking of relative cell edge movement can be performed with less than 40 nm precision. The percentage change in cell volume was computed from the relative deformations (cellular strains) along the cell major and minor axis, assuming the rotational symmetry of a prolate spheroid, that is, 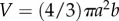, where *b* and *a* represent the lengths of cells along major and minor axis, respectively. For each optical stretcher experiment, the number of collected cells was *n* ≥ 30. The cellular strain and compliance data are presented as mean ± s.e.m. Representative strain and compliance data were chosen from two or more independent experiments. In order to correct for different cellular response owing to slight variations in cell cycle or nutrient concentration in a particular batch of medium (e.g. HL60 cells have been reported to show decreased strain with increased culture density [14]), data for each power were taken over a number of days. To minimize additional systematic errors, for example changes in cell deformability with post-incubation period [30], cells were stretched with a random sequence of powers for each experiment. During stretching, a range of cell sizes were measured to ensure the results were representative of the entire population. Care was taken to exclude any irregular-shaped cells, as they introduce unwanted rotations during stretching, giving rise to ‘false’ deformations. The flow was adjusted and always made to stop before trapping to minimize rotations and wobbling before the start of a stretch. To avoid non-uniform pressure gradient that disturbs the flow, care was taken to remove any air bubbles in the capillary and cell debris in suspension. The latter was minimized by using rapidly growing cells (logarithmic phase) for experiments or centrifuging cells before experiment.

### 2.3. Cell preparation

HL60/S4 myeloid precursor cells were chosen as the model cells for this study, because they naturally grow in suspension, which means they are measured in their physiological environment in a microfluidic optical stretcher. The cells were incubated at 37.5°C with 5% carbon dioxide level. Cells were chosen to be stretched when they were at their logarithmic phase of growth, which occurred typically 36–48 h after resuspension. Trypan blue exclusion method was employed to check for cell viability prior to every experiment. Cells were kept incubated in vials and allowed to equilibrate at a specific chamber temperature for 20 min prior to optical stretching experiments. All optical stretching experiments were performed within 2 h after the cells were taken out of the incubator. For calcium imaging experiments, HL60 cells were loaded with 1 μM Fluo-4, AM (Invitrogen, F14201) and incubated for 20 min at 25°C. Subsequently, the AM ester solutions were removed by centrifugation and cells were resuspended in RPMI 1640 medium or phosphate buffered saline (PBS) medium without calcium, unless otherwise stated. For experiments on inhibiting TRPV2 ion channels, cells were measured in 10 μM ruthenium red (Sigma-Aldrich, 84071) solution.

## 3. Results

### 3.1. Cells are more compliant at higher temperatures

**Figure 2.**
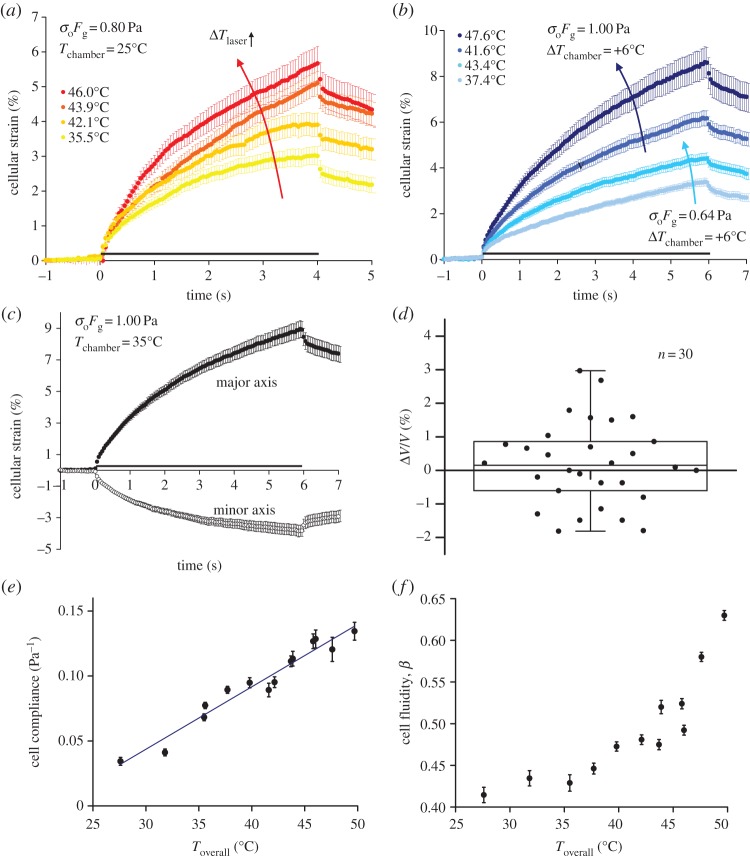
Passive viscoelastic cell deformation in response to heating. (*a*) Cellular strain as a function of time when the cells were stretched at 0.80 Pa by the 1064 nm laser and heated instantaneously to higher temperatures by the 1480 nm heating laser with chamber temperature kept at 25°C. The red arrow indicates increasing temperature owing to laser heating. (*b*) Cellular strain as a function of time when the cells were stretched at 0.64 and 1.00 Pa but subjected to an increase in the chamber temperature. The blue arrows indicate increasing temperature owing to chamber heating. (*c*) Cellular strain along the major and minor axes as a function of time when the cells were stretched at 1.00 Pa and 35°C. (*d*) Change in cell volume after 6 s of stretching. Data represent cells stretched at *T*_overall_ < 52°C. Mean change in cell volume = 0.21% with s.e.m. = 0.23%. (*e*) Cell compliance at 4 s into the stretch, *J*(*t* = 4 s), plotted as a function of overall temperature 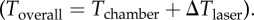 The line represents a linear fit to the data points, with *R*^2^ = 0.95. (*f*) Cell fluidity, *β*, plotted as a function of *T*_overall_. Legends in (*a*,*b*) indicate *T*_overall_. Grey bars along the time axis in (*a–c*) indicate the period of stretch.

To investigate the effect on cell deformation as it experiences a sudden temperature jump, we conducted optical stretching experiments using the 1480 nm laser set-up, where an instantaneous temperature jump within milliseconds was applied in addition to the deformation by the 1064 nm stretch laser, as described in §2.1. Using the calibrated temperature increase for heating by the 1480 nm laser, we observed an increase in peak cellular strain along the cell's major axis (parallel to the laser axis) with increased laser heating 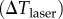, as indicated by the red arrow in figure 2*a*. To investigate whether a similar thermal softening behaviour is observed for cells under prolonged exposure to heat, we stretched cells with the same optical stress but subjected to higher chamber temperatures. Figure 2*b* compares the strain curves for cells stretched at 0.64 and 1.00 Pa, each subjected to an increase in chamber temperature from 29 to 35°C. Clearly, the peak strain increased with increased chamber temperatures at both stresses. Further experiments at higher stresses with increased chamber temperatures revealed the same thermal softening behaviour (not shown).

The temporal evolution of cellular strain along the minor axis was also characterized (figure 2*c*, open circles). The cells showed a negative strain in this direction, with the calculated Poisson ratios very close to 0.5. This suggests that the cell volume was conserved, assuming that the cells remained rotationally symmetric in the form of a prolate spheroid during the stretch. Indeed, no significant change in cell volume was observed (figure 2*d*) as long as the overall temperature stayed below 52°C. To compare changes to cell material properties at different overall temperatures, we computed the cell compliance at 4 s into the stretch *J*(*t* = 4 s) using equation (2.1), and the results are plotted in figure 2*e*. Clearly, the cell compliance increased linearly with the overall temperature, suggesting that the cell compliance is only a function of the absolute overall temperature, independent of the time scales involved in the heat treatments. The thermal softening effect is significant, with the cells experiencing a marked fourfold increase in creep compliance as the temperature increased from 27 to 47°C. The cell fluidity, *β*, can also be obtained by fitting the creep compliance curves to the power-law model from equation (2.2), and the results are summarized in figure 2*f*. Here, we observed a low value of *β* hovering below 0.45, when the overall temperatures were below 37°C. Above the physiological temperatures, there appears to be an onset of a nonlinear increase in the cell fluidity, suggesting some form of a multiphasic response. To conclude, in the presence of higher temperatures induced by a sudden temperature jump or prolonged exposure to heat, the cells were able to adapt and remodel themselves to become more mechanically compliant and fluid-like.

### 3.2. Cells actively contract above a critical temperature

**Figure 3.**
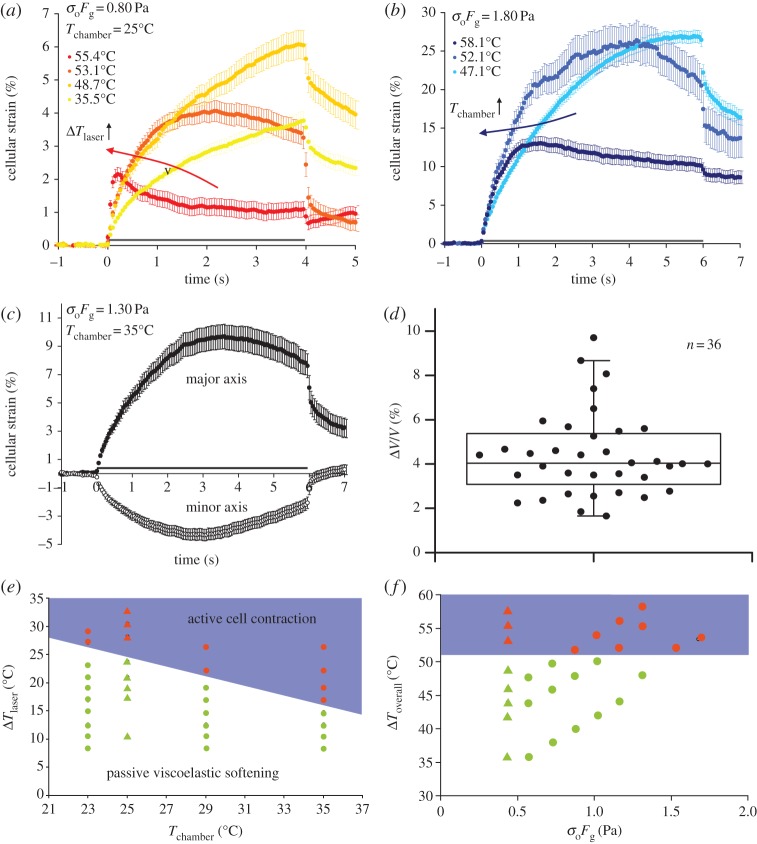
Active cell contraction in response to heating. (*a*) Cellular strain as a function of time when the cells were stretched at 0.80 Pa and heated by the 1480 nm heating laser to *T*_overall_ above 52°C. The red arrow indicates increasing temperature owing to laser heating. (*b*) Cellular strain as a function of time when the cells were stretched at 1.80 Pa and subjected to higher chamber temperatures such that *T*_overall_ exceeds 52°C. The blue arrow indicates increasing temperature owing to chamber heating. (*c*) Cellular strain along the major and minor axes, as a function of time, at 1.30 Pa and 35°C, which corresponded to *T*_overall_ = 52°C. (*d*) Change in cell volume after 6 s of stretching. Data represent cells stretched at *T*_overall_ ≥ 52°C. Mean change in cell volume = 4.39% with s.e.m. = 0.31%. (*e*) Schematic phase diagram showing cellular response when the cells were subjected to an ambient combination of laser-induced temperature increase 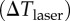 and chamber temperature (*T*_chamber_). Triangular symbols denote cellular response by the 1480 nm heating at 0.80 Pa at *T*_chamber_ = 25°C. Circular symbols denote cellular response when stretched and heated by the 1064 nm laser only. (*f*) Schematic phase diagram showing cellular response when the cells were subjected to various optically induced stresses (*σ*_0_*F*_g_) at various *T*_overall_. Legends in (*a*,*b*) indicate *T*_overall_. Grey bars along the time axis in (*a*–*c*) indicate the period of stretch.

Interestingly, we observed a strange, unexpected cellular response at much higher overall temperatures, independent of the time scales of heat treatments. In the first set of experiments, we heated up the cells with various powers of 1480 nm heating laser for 4 s, before stretching them at a constant stress of 0.80 Pa. The results of cellular deformation (along cell major axis) against time at various overall temperatures are shown in figure 3*a*. The initial increase in cellular strain at higher temperature (yellow to gold) was indicative of thermal softening behaviour, as discussed in the previous section, but at even higher temperatures the cells began to exhibit contractile behaviour along the major axis (orange and red). The transition temperature was observed to occur at around 52 ± 1°C. To further investigate whether the observed contraction was indeed induced by heat and occurred only at this particular temperature, we carried out optical stretching experiments where the temperature increase was adjusted through raising the chamber temperature. As shown in figure 3*b*, cells showed normal viscoelastic deformation (light blue) when first stretched at 1.80 Pa at a chamber temperature of 23°C. When the cells were stretched at the same stress but at higher chamber temperatures of 29 and 35°C (medium and dark blue), we observed an initial deformation followed by a contraction along the cell major axis. The same transition to contraction at elevated chamber temperatures was also observed for other stretch powers. Using the predetermined calibrated temperature increase (10.5 ± 1°C W^−1^ for 1064 nm stretch laser), we arrived at the same observation that cell contraction occurred only when the overall temperature rose above 52 ± 1°C. We note that the results were striking given that the same active contraction was observed independent of how the cells were heated, specifically the time scales involved.

The temporal evolution of cellular strain along the minor axis (figure 3*c*, open circles) showed that the negative strain rate did not extend throughout the stretch. Instead, it reached a peak negative strain before assuming a positive rate of strain. The peak strains along both the major and minor axis occurred at around the same time (approx. 3–4 s). Interestingly, despite its contraction along the major axis, the cell expanded in volume. The change in cell volume throughout the stretch was significant (4.39 ± 0.31%), as shown in figure 3*d*, which is in contrast to the non-contracting cells which underwent normal viscoelastic deformation at lower overall temperatures (figure 2*d*).

We followed up with a series of experiments to systematically investigate the cellular response over a range of overall temperatures achieved through various combinations of laser-induced heating and adjustment of chamber temperatures. The results are depicted in a schematic phase diagram (figure 3*e*) with the space spanned by two parameters 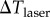 and *T*_chamber_ denoting laser-induced temperature increase and the chamber temperature, respectively. Notably, we observed active cell contraction (orange symbols, blue region) whenever the overall temperature, independent of the combination of chamber temperature or laser-induced temperature increase, rose above 52°C. On the other hand, when the overall temperature was below 52 ± 1°C (green symbols, white region), we observed only passive, thermal softening behaviour characterized by an increase in cell compliance and fluidity with increased overall temperature. We therefore termed this region as active cell contraction to distinguish this behaviour from the passive viscoelastic softening behaviour at lower overall temperatures.

To determine whether mechanical stress plays a role in dictating the peculiar response of cells at elevated temperatures, we mapped the cellular response in another schematic phase diagram spanned by the overall temperature *T*_overall_ and the optically induced stress *σ*_0_*F*_g_. As shown in figure 3*f*, the transition from passive viscoelastic deformation to active cell contraction at 52°C depends only on the overall temperature but not the magnitude of optically induced stress exerted by the 1064 nm stretch laser. This suggests that the observed active contraction was triggered by a heat-sensitive mechanism instead of a specific response to a large mechanical stress. More specifically, this implies that the cell actively contracts regardless of the amount of axial strain it experiences, as shown in figure 3*a* where contraction was seen to occur at very small peak strain (4%).

### 3.3. Temperature-sensitive TRPV2 ion channels regulate cell mechanical response

**Figure 4.**
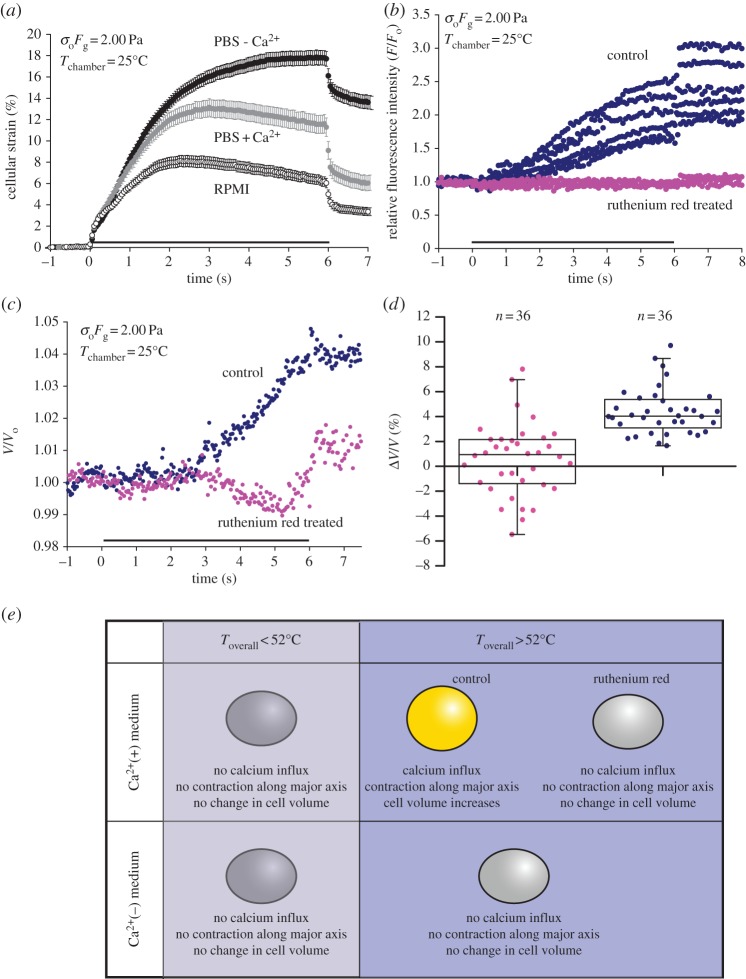
Mechanism of thermomechanical response. (*a*) Cellular strain as a function of time when the cells were stretched with 2.00 Pa at 25°C, in PBS (with and without calcium) and RPMI. (*b*) Relative fluorescence intensity (*F*/*F*_0_) for representative control cells (blue) and cells treated with ruthenium red (magenta), as a function time, when loaded with Ca^2+^ dye Fluo-4 and stretched in calcium-rich PBS at *T*_overall_ ≥ 52°C. (*c*) Change in cell volume for a representative control cell (blue) and a cell treated with ruthenium red (magenta), when stretched at *T*_overall_ ≥ 52°C. (*d*) Box plots showing the change in cell volume for control cells (blue) and cells treated with ruthenium red or in calcium-free medium (magenta) at *T*_overall_ ≥ 52°C. The two populations are significantly different (*p* < 0.01). (*e*) Schematic diagram of cellular response in different buffer conditions above and below 52°C. Grey bars along the time axis in (*a–c*) indicate the period of stretch.

Motivated by a recent paper on the role of calcium in regulating cell mechanics at elevated temperatures [23], we hypothesized that calcium influx could perhaps lead to enhanced myosin activity and the observed cell contraction above 52°C. To test this hypothesis, separate experiments were performed in different media, namely PBS with or without free calcium ions and in RPMI 1640 medium enriched with 0.42 mM of free calcium ions. The results of the experiments are shown in figure 4*a*. When the cells were stretched with 2.00 Pa at 25°C, which corresponds to an overall temperature of 52°C, cell contraction was only observed in the presence of extracellular calcium ions, indicating a crucial role of calcium in cell contraction. The difference in refractive index between PBS and RPMI is small (*n*_PBS_ = 1.334, *n*_RPMI_ = 1.338), so the difference in optically induced stress on the cells in both media is negligible. Furthermore, the measured osmolalities for PBS with calcium (282 mOsm kg^−1^ H_2_O) and without calcium (300 mOsm kg^−1^ H_2_O) are comparable, so the change in cellular response is not likely to be a result of a change in osmotic condition.

We speculated that the observed cellular contraction must be attributed to calcium signalling. As such, we performed calcium imaging experiments on cells loaded with the Ca^2+^ dye Fluo-4. Images of the fluorescent cells can be found in the electronic supplementary material, figure S2. When we stretched the cells with 2.00 Pa at 25°C, which corresponds to an overall temperature of 52°C, we observed a pronounced increase in fluorescence intensity in the cell (figure 4*b*, blue), though no significant change in fluorescence intensity was observed for cells stretched below this critical temperature. This indicates that cell contraction may be caused by calcium influx triggered by heat. A plausible mechanism may be owing to the activation of temperature-sensitive TRPV2 ion channels which have been reported to have the highest opening probability at approximately 52°C [31,32]. Expression of such proteins in HL60 cells has also been positively confirmed in the past [33,34]. To this end, we added 10 μM of ruthenium red to the cells prior to stretching. Ruthenium red has been reported to be an effective blocker of TRPV2 channels [35]. When stretched with an overall temperature of 52°C, we observed only a low baseline fluorescence in the cell (figure 4*b*, magenta), indicating that no measurable amount of Ca^2+^ entered the cell. Simultaneous imaging of cell deformation also showed normal deformation without contraction. This suggests that TRPV2 channel activation is the primary pathway for calcium influx and regulates the thermomechanical response of HL60 cells. When we performed the same optical stretching experiments under the same experimental conditions but in calcium-free PBS medium, cells again displayed only low baseline fluorescence throughout the stretch (similar to figure 4*b*, magenta), indicating that the observed increase in fluorescence intensity was very likely owing to the influx of extracellular calcium ions and not through intracellular release via internal stores.

Apart from driving cell contraction, the influx of calcium ions could generate an osmotic potential that drives the inflow of water, leading to the subsequent expansion of cell volume, as shown for a representative contracting cell in figure 4*c* (blue). Specifically, there was a time lag between the onset of calcium influx which occurred immediately after the stretch at *t* = 2 s and the onset of volume expansion (*t* = 4 s). Interestingly, cells treated with ruthenium red showed no contraction along the major and minor axes and no significant change in cell volume (magenta) throughout the stretch period. Further analysis for a sample population of cells for both cases is depicted in figure 4*d*. The contracting cells expanded significantly (4.85 ± 0.60%) after the stretch, while no significant volume change was observed for cells treated with ruthenium red or immersed in calcium-free medium (0.56 ± 0.48%). The picture emerging from our results is schematically summarized in figure 4*e*. Our data suggest a strong correlation between the three variables. While the calcium influx, through the activation of TRPV2 ion channels, may directly impact cell contraction along the cell major axis, it also correlates with overall cell expansion in its volume, albeit a delayed response. To conclude, we have identified TRPV2 ion channels as a crucial player in regulating cell mechanics and cell-shape changes in response to external heat in the presence of extracellular calcium ions.

### 3.4. Theoretical modelling of thermorheological behaviour

**Figure 5.**
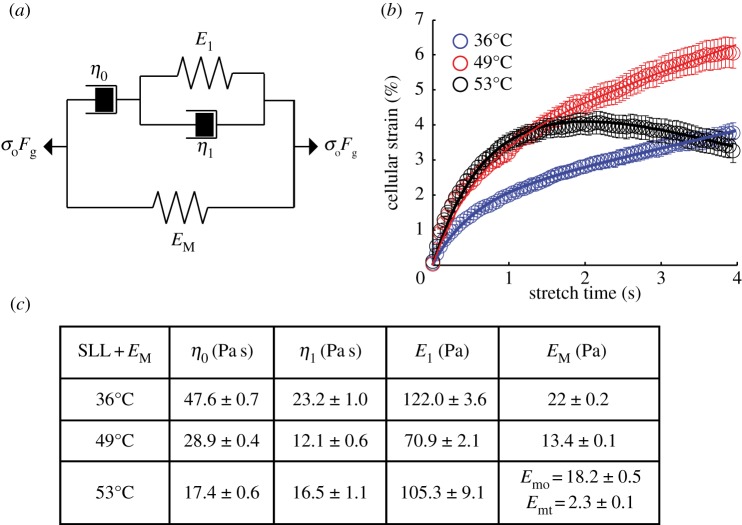
Mechanically equivalent circuit describing the thermomechanical regulation of HL60 cells. (*a*) The model consists of a SLL model in parallel with a dynamic elastic component *E*_M_ arising from cell cortical tension. *σ*_0_*F*_g_ denotes the constant optically induced stress. (*b*) Theoretical creep response of the mechanical model predicting a transition from passive viscoelastic deformation (open circles: experiment; blue line: model fit) to thermal softening (open circles: experiment; red line: model fit), followed by active cell contraction above 52°C (open circles: experiment; black line: model fit). The legend indicates the overall temperatures. (*c*) Various viscoelastic parameters extracted from fitting the mechanical model to the cell deformation curves at low and high temperatures.

The rheological transition from thermal softening to active cell contraction can be described by a mechanically equivalent model (figure 5*a*), which essentially consists of a SLL component in parallel with an elastic element *E*_M_(*t*) originating from the effective cell elasticity generated by the cell cortical tension *T*. Indeed, the deformation of the cell from its initial spherical shape leads to an expansion of the cell surface resisted by its surface tension. Assuming that the optical stretcher applied a pressure profile *σ*(*θ*) = *σ*_0_cos^2^*θ*, where *θ* is the meridional angle in a polar coordinate where the polar axis is aligned with the laser optical axis, and furthermore assuming cell volume conservation, one finds an effective elastic modulus 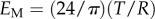 resisting cell deformation, with *R* the cell radius. In addition, *η*_0_ represents the effective cellular long time-scale viscosity, possibly originating from molecular slip between the transient cross-linkers in the cytoskeleton, *η*_1_ represents the transient viscous effect possibly arising from the poroelastic effect [36], while *E*_1_ represents the elastic response within the cytoskeleton. Because *E*_M_ is associated with the cell cortical tension generated mainly by the myosin II activity, it is expected to be significant at a long time scale compared to cytoskeletal turnover events [37]. We numerically solved the equations of the mechanical system under constant external force at temperatures below and above the critical temperatures (see the electronic supplementary material).

We assumed that at low temperatures when the cells experienced no calcium influx during the short period of stretch (4 s), the effective elastic modulus *E*_M_ remained approximately constant during the stretch. In this case, the predicted creep response consisted of three regimes: a very fast elastic response, a slowing down of the deformation followed by a viscous flow, in line with the experimental observation. A numerical fit to the strain curves at 36 and 49°C, as shown in figure 5*b* (blue and red lines), allowed us to extract the various viscoelastic parameters as tabulated in figure 5*c*. Our results predicted two changes to cell material properties at higher temperatures. First, there is a drop in the steady-state viscosity *η*_0_, indicative of a more latent fluid-like behaviour at long time scales at higher temperatures. Second, there is a concomitant decrease in *E*_M_, suggesting that at a higher temperature, the drop in cell compliance is also associated with a reduction in cell cortical tension.

Above the critical temperature when the TRPV2 ion channels get activated, we assume that the dominant effect of calcium influx is the formation of calcium–camodulin complexes which triggers extensive myosin phosphorylation and generates large cell cortical tension [38,39]. We assume that this leads to a time-dependent, effective elastic modulus *E*_M_(*t*) with a temporal profile that is linearly proportional to the calcium influx indicated by the average fluorescence intensity *I*(*t*) (representative curves are depicted in figure 4*b*). Specifically, this leads to *E*_M_(*t*) = *αI*(*t*) = *E*_mo_ + *E*_mt_(*t*/*τ*_0_)*^*γ*^*, where *τ*_0_ is assumed to be 1 s and *E*_mo_ and *E*_mt_ represent the static and dynamic terms, respectively. The exponent *γ* follows from *I*(*t*), which was found to scale with a power-law form, *I*(*t*) ∼ *t^*γ*^* with *γ* = 1.298 ± 0.062. Incorporating this final form of *E*_M_(*t*), the numerical solutions predict a transition from an initial viscoelastic deformation to a subsequent contraction within a few seconds (figure 5*b*, black), in agreement with our experimental observations of a delayed response between the initial calcium influx and the effect of phosphorylation. Furthermore, by performing a good fit to the strain curve at 53°C, as shown in figure 5*b* (black line), we are able to extract all the five viscoelastic parameters, as shown in figure 5*c*.

## 4. Discussion

Our results demonstrate clearly that an increase in ambient temperatures in and around a suspended cell can significantly impact its material properties, making it more mechanically compliant and fluid-like. Previous studies [17,19,40,41] observed similar thermal softening behaviour using a number of techniques on both adherent and suspended cells, such as fibroblasts and leucocytes. Interestingly, other studies indicated the opposite effect of thermal stiffening with adherent cells grown on culture dishes [20,42], possibly owing to increased contractile activity of molecular motors leading to enhanced cellular prestress and stiffness. This was not the case in our measurements, where cells assumed a round geometry in their naturally suspended state in the absence of stress fibres. The increase in cell compliance with higher temperatures has also been confirmed with another recent independent study using the optical stretcher [24], where the authors explained the qualitative change in the thermorheology of cells by invoking the concept of time–temperature superposition widely used in polymer physics. Despite the similarity of our work, we further characterized the changes to suspended cell fluidity with temperature variations, which is, to our knowledge, the first systematic study in this area.

The observed creep compliance of cells appeared to scale linearly with the overall temperatures (figure 2*e*). Previous studies observed an increased dissociation rate of protein cross-linkers, for example *α*-actinin, with temperature [43]. *In vitro* studies have shown that such thermal unbinding of transient cross-links results in stress relaxation which decreases the static network elasticity and increases the viscous dissipation [44,45]. Based on the affine stretching model [46], such mechanism predicts an inverse temperature dependence of network elasticity 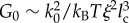, where *k*_0_ is the actin filament bending stiffness, *ξ* is the network mesh size and *l*_c_ is the average cross-linker distance. This translates to a linear temperature dependence of creep compliance, in good agreement with our observation. The increase in viscous dissipation owing to thermal unbinding of transient cross-linkers may also explain the observed changes to the viscoelastic parameters in both models at higher temperatures: the increase in cell fluidity *β* (figure 2*f*) within the power-law model, and the decrease in both the steady-state viscosity *η*_0_ and the effective elastic modulus *E*_M_, which originated from the cell cortical tension, within the mechanical model (figure 5*c*). At 36°C, our theoretical model predicted *E*_M_ ∼ 22 Pa, which is equivalent to a cortical tension of 25 pN μm^−1^, close to that of blood granulocytes (30 pN μm^−1^) as measured with the micropipette aspiration technique [47,48]. Furthermore, our theoretical model predicted a drop in cell cortical tension at higher temperatures, consistent with recent experimental studies [40,41,49]. Our results therefore highlight the fundamental role that cell cortical tension plays in resisting cell deformation, the reduction of which leads to a significant increase in the cell compliance.

At significantly higher temperatures, our results revealed a dramatic change from passive deformation to an active cell response regulated by the TRPV2, which provides an ion channel gating mechanism for cellular entry of calcium ions. TRPV2 ion channels have recently been shown to play a crucial role in early steps of phagocytosis [35] and regulatory volume decrease [50]. Our work further suggests that these temperature-sensitive ion channels help to regulate the cell contractility through coupled dynamics of calcium influx and increased myosin activity. Myosin II, an important molecular motor, is known to play a crucial role in cell contractility and shape control [37]. One previous study [51] observed reversible motor activity without denaturation upon raising the temperature instantaneously to above 60°C. On a similar note, Wetzel *et al*. [22] showed that when the cells were exposed to less than 3 s of laser heating in an optical stretcher with overall temperature above 50°C, more than 70% of the stretched cells remained viable. We note that the observed cell contraction was rapid, typically 2 s following the onset of calcium influx (figure 4*b*,*c*). This suggests that the signalling mechanism was possibly a first-order event, for example an increase in myosin phosphorylation via the calmodulin-myosin light chain kinase (MLCK) pathway, which regulates the assembly of myosin motors into contractile filaments, leading to subsequent cell contractility [38,39,52,53]. The pathway involves the binding of calmodulin to four calcium ions and the MLCK and the typical time scales of this reaction are less than a second. We simulated this process with a mechanical model which includes a dynamical elastic term *E*_M_(*t*), which grows in time in response to the transient calcium influx, and successfully predicted a transition from passive deformation to active cellular contraction. Our results also suggested that in the active regime, the cellular response is dominated by the increased myosin activity owing to calcium influx, rather than the thermal unbinding mechanism of transient cross-linkers, which accounts for cell softening in the passive regime. Further experiments on cells treated with myosin inhibitors, for example blebbistatin, in the active regime showed a partial abolishment of the active contraction to the point where the strain rate of the major axis never became negative (see electronic supplementary material, figure S3), indicating that Ca^2+^-activated myosin activity is indeed involved in active cell contraction, though other mechanism(s) may be involved as well. Our results are consistent with some of the latest findings by Gyger *et al*. [54] where they observed similar active contractions in suspended epithelial cells transfected with TRPV channels. Nevertheless, contrary to their report, we observed no negative cellular strain during active cellular contractions within the time scale of 6 s. More importantly, while Gyger *et al*. adopted a phenomenological mathematical model to account for active cellular contractions, our mechanical model offers a simpler, more intuitive approach, which sheds light on how active remodelling of cell cortical tension can regulate cell-shape changes in response to heat and biochemical signals.

The observed time scale for an osmotic response is in agreement with previous reports on diffusion dynamics of water across cell membranes (diffusion time approx. 0.2 s) [55,56]. Assuming that the cell volume changes only by the permeation of water through the cell membrane, the water flux is given by the classical permeation law 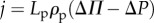, where *L*_p_ is the permeation constant of one pore, *ρ*_p_ is the pore density in the membrane, 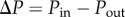 is the hydrostatic pressure difference between inside and outside of the cell and 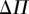 is the osmotic pressure difference between inside and outside of the cell. At quasi-static equilibrium, the relative change in cell volume is proportional to the relative change in intracellular osmolyte concentration [57]. Assuming that the calcium influx contributes only passively to the cell osmotic pressure, the observed increase in cell volume (approx. 5%) suggests that the amount of calcium entering through calcium channels represents roughly 5% of the total number of osmolytes in the cell.

We rule out other potential mechanisms for cell contraction. Firstly, it is likely that an increase in intracellular water content may lead to a potential drop in cell refractive index, and consequently the optically induced stress, as the latter is proportional to the difference in refractive index between the cell and the medium [14]. This reduction in optical stress could lead to a drop in cell deformation, and hence the observed ‘contraction’, which would then be understood as a reduction in the stretching force. We argue that this increase in cell volume was too small to initiate any significant change in overall cell refractive index, as supported by Kemper's study [58] on human pancreas and liver tumour cells, which showed an approximate 0.36% drop in cell refractive index with a 4% increase in cell volume. A similar drop in cell refractive index with increase in cell volume has also been reported recently in macrophages [59]. Secondly, we exclude the role of stretch-activated ion channels in regulating cell contraction, as contraction was only dependent on the overall temperature but not the magnitude of optically induced stress and the resultant peak strain (figure 3*f*). Specifically, active cell contraction can occur at very low peak strain (4%) when heated by 1480 nm laser (figure 3*a*). Finally, it is plausible that below 52 ± 1°C contraction was already taking place but was suppressed by dominant passive deformation, and one could argue that cells may contract given sufficient time of observation. We ruled out such a possibility, as we observed no active contraction below 52°C, even when the cells were stretched for 30 s (data not shown). Nevertheless, these mechanisms could still contribute or modify the actually observed cell behaviour, but the dominant effect is likely to be the increase in myosin activity in response to the calcium influx through TRPV2 ion channels above 52 ± 1°C.

## 5. Conclusion

In this work, we introduced a modified version of a microfluidic optical stretcher with integrated heating components, which allowed us to probe cell mechanics when the cells were subjected to an extended period of heating or to a sudden increase in temperature. We find that the cell compliance of HL60 cells scales linearly with the temperature, independent of the time scales of thermal treatments, and exhibits more fluid-like behaviour at higher temperatures. Our results suggest that in the passive viscoelastic regime below a critical temperature, the rheology of HL60 cells can be effectively described by the thermal unbinding mechanism of transient cross-links in the actin cortex. Beyond a critical temperature of 52 ± 1°C, the cells exhibit active contraction along the direction of maximal stress owing to the activation of TRPV2 ion channels, leading to an eventual expansion in overall cell volume. Our work points to an important role of TRPV2 ion channels and calcium signalling in the active regulation of cell cortex stiffness and cell-shape changes at elevated temperatures.

## Funding statement

The authors are grateful to the EU Marie Curie Initial Training Networks TRANSPOL (to C.J.C.) and the Engineering and Physical Sciences Research Council (to L.B.) for funding this project through scholarship support. J.G. acknowledges financial support via the ‘LightTouch’ Starting Investigator Grant of the European Research Council and the Humboldt Professorship from the Alexander von Humboldt Foundation. The authors also acknowledge financial support by the Human Frontier Science Program (to J.G. and G.W.) and the Cluster of Excellence EAM at FAU Erlangen-Nuremberg funded by the German Research Foundation (DFG) in the framework of its Excellence Initiative (to G.W.). The HL60/S4 cell line was a generous gift from Don and Ada Olins, University of New England.
